# In Vitro Biocompatibility Evaluation of Nine Dermal Fillers on L929 Cell Line

**DOI:** 10.1155/2020/8676343

**Published:** 2020-05-21

**Authors:** Vincenza Cannella, Roberta Altomare, Vincenza Leonardi, Laura Russotto, Santina Di Bella, Francesco Mira, Annalisa Guercio

**Affiliations:** ^1^Istituto Zooprofilattico Sperimentale della Sicilia “A. Mirri”, Via Gino Marinuzzi, 3–90129 Palermo (PA), Italy; ^2^Department of Psychology, Pedagogy and Sports Sciences, AOUP Paolo Giaccone, University of Palermo, Italy

## Abstract

**Objective:**

Biomaterial research for soft tissue augmentation is an increasing topic in aesthetic medicine. Hyaluronic acid (HA) fillers are widely used for their low invasiveness and easy application to correct aesthetic defects or traumatic injuries. Some complications as acute or chronic inflammation can occur in patients following the injection. Biocompatibility assays are required for medical devices intended for human use, in order to prevent damages or injuries in the host. In this study, nine HA fillers were tested in order to evaluate their cytotoxicity and their effects on L929 cell line, according to the UNI EN ISO 10993 regulation.

**Methods:**

Extracts were prepared from nine HA fillers, and MTS viability assay was performed after 24 h, 48 h, and 72 h of exposure of cells to extracts. Cells cultured with HA filler extracts were monitored for up to 72 h, counted, and stained with haematoxylin/eosin in order to evaluate the cell proliferation rate and morphology.

**Results:**

None of the filler tested showed a cytotoxic effect. Two samples showed a higher vitality percentage and higher cell number while two samples showed a lower vitality percentage and lower cell number at 72 h.

**Conclusion:**

Data obtained suggest that although examined fillers are not cytotoxic, they show different effects on the *in vitro* cell proliferation rate. *In vitro* studies of medical devices could lead to important implications since these could aid to predict effects about their *in vivo* application. These easy and rapid assays could be useful to test new materials intended for human use avoiding animal tests.

## 1. Introduction

In the last few years, biomaterial research for soft tissue augmentation has been implemented for its application for the correction of serious and slight aesthetic defects or traumatic injuries [1]. Injectable fillers are widely used for their low invasiveness and easy application. Fillers, as medical devices, must be biocompatible by inducing an appropriate biological response in a specific application, without causing damage or injury [2]. This involve an interaction between the host environment, the material, and the function that it must perform. Biocompatibility is a dynamic process aimed at minimizing any adverse reaction or rejection by the host [3]. Since a filler integrates with the body, skin, and derma, the interaction of the material with the surrounding tissues could be responsible of many biological reactions. Based on their chemical composition, they can be classified into “biological fillers” and “synthetic fillers”. The first ones consist on natural molecules that undergo skin resorption after a certain period of time. They are represented by collagen, hyaluronic acid, and polylactic acid. The “synthetic fillers” are derived from synthesis processes, and their effect is more prolonged compared to biological fillers. The most common synthetic fillers are polyacrylamide, polymethylmethacrylate, polytetraethylene, and povidone. Therefore, according to their longevity, fillers can be permanent or temporary. The main advantage from the use of fillers is obtaining harmonious and natural results immediately, without resorting to invasive surgical procedures. However, acute inflammatory reactions are the most frequent complications that occur immediately following the injection. Other complications are granulomas, herpes labialis, permanent redness, small necrosis, or abscesses. Chronic inflammations could occur after a certain period [4, 5]. Hyaluronic acid (HA) is one of the most common naturally derived filler, and, nowadays the most frequently used, even if its short persistence, possible allergenicity, and immunogenicity leads to the research of new synthetic materials [5]. HA is an anionic, nonsulphated glycosaminoglycan widely represented in connective, epithelial, and neural tissues. HA is one of the major components of the skin, and it is involved in tissue regeneration, due to its high water-binding properties, maintaining proper tissue volume. Impair of HA promotes biological ageing and wrinkle development. A variety of HA-based fillers has been approved for the treatment of wrinkles, scars, and facial contouring defects [6]. HA used to produce fillers is synthesized in laboratory from bacterial cultures. In order to guarantee an adequate permanence once injected into the tissues, HA chains undergo a cross-linking process with linking substances, which ensure a greater resistance to the action of enzymes in the body. One of the most used linking substances is BDDE (1,4-butanediol diglycidyl ether) [5].

HA dermal fillers are classified into two categories, monophasic or biphasic. The monophasic ones consist of solid particles of material, plunged in a fluid carrier substance, and appear as a homogeneous gel. Biphasic fillers are composed of cross-linked HA particles immersed in a fluid matrix consisting of low or zero cross-linked HA. Monophasic HA fillers are more cohesive, may last longer, and show a low migration rate after injection; biphasic HA fillers are more easily customized and adaptable to the anatomical area being treated [7].

A filler must be similar to the native tissue, durable, easily implantable, and painless for the patient. Moreover, it should be nontoxic, noncarcinogenic, inert, nonallergic, nonimmunogenic, nonpyrogenic, and nonmigrating [2]. For this reason, marketing of any device, intended for human use, requires the assessment of the biological response [8]. The evaluation of cytotoxicity is a crucial step to establish the biocompatibility of a material [9, 10]. The European Directive 63/2010/EU, defines the concept of 3Rs (*Replacement*, *Reduction* and *Refinement*) addressing the interest of the scientific community towards the application of *in vitro* methods as alternatives approach to the *in vivo* methods [11]. The interaction of cell cultures with potential toxic compounds released by a biomaterial induces a detectable biological response through which it is possible to establish the safety of a material under examination. Cytotoxicity tests allow to highlight the possible alterations in basic cellular functions through the analysis of cellular metabolism, morphology, and the proliferation rate or vitality [12]. The UNI EN ISO 10993/2009 rule, part five, describes suitable *in vitro* methods to perform the biological evaluation of medical devices [13]. Tests can be performed through a direct or indirect contact of cells with the material or through a contact with an extract, depending on the nature and the shape of the material under examination. [14–16]. Medical devices, including fillers for aesthetic medicine, are not subject to any prior authorization from the Competent Authority (Ministry of Health), as there is a European regulation of “new approach”, according to which medical devices are certified by notified bodies authorized in the European Country [17].

This study is aimed at evaluating the biocompatibility of nine commercially available different HA fillers by performing a cytotoxicity test and cell proliferation test on the L929 cell line.

## 2. Materials and Methods

### 2.1. Samples

Samples used were nine different HA fillers commercially available with an average content of 20 mg/ml HA. Filler samples arrived in the laboratory sealed in their packaging, and sterility was guaranteed by the manufacturer. More detailed characteristics of each filler are described in Table 1.

### 2.2. Cell Culture

L929 cell line (murine fibroblast) was purchased from Cell Bank of National Reference Institute for Alternative Methods, Welfare and Care of Laboratory Animals (Istituto Zooprofilattico Sperimentale della Lombardia ed Emilia Romagna, Italy). Cells were grown in culture flasks containing minimum essential medium (MEM, Sigma-Aldrich), supplemented with 10% fetal bovine serum (FBS, Euroclone), 1% antibiotic-antimycotic solution (Sigma-Aldrich), and 1% nonessential amino acids (NEAA, Euroclone). Cells were maintained at +37°C in a humidified 5% CO_2_ atmosphere and monitored daily by using an inverted microscope. Subcultures were performed twice a week, when an 80% of confluence was observed.

### 2.3. Sample Preparation

The “extraction dilution method” was chosen as described by the UNI EN ISO 10993 regulation [13]. Extraction procedure was carried out in an extraction medium consisting of MEM, supplemented with 10% FBS, 1% antibiotic-antimycotic solution, and 1% NEAA at +37°C ± 1 for 24 h, by continuous agitation. An amount of 0.2 g of each filler was dissolved in 1 ml of extraction medium. The extraction medium without sample was used as reagent control (RC) and treated as a sample. A 5% phenol solution was used as positive control (PC).

### 2.4. Cytotoxicity Test on the L929 Cell Line

Cells were seeded into 96-well culture plates at 1 × 10^5^ cells/ml ratio in MEM, supplemented with 10% FBS, 1% antibiotic-antimycotic solution, and 1% NEAA. Three 96-well culture plates for each filler were prepared and incubated at +37°C ± 1 in 5% CO_2_ for 24 h. After this time, culture media were replaced with 100 *μ*l of each filler extract and the control extracts and a series of twofold dilutions (from 100% to 3,125% concentrations). The assay was carried out in triplicate. Moreover, intralaboratory assays were performed. Some wells were filled with MEM, supplemented with 10% FBS, 1% antibiotic-antimycotic solution, and 1% NEAA, and used as control cells (negative control). All plates were incubated at +37 ± 1°C in a 5% CO_2_ atmosphere and examined microscopically after 24 h, 48 h, and 72 h of incubation in order to assess vitality and general morphology of cells. The vitality MTS assay was performed at the same time points as previously described [12]. The absorbance recorded is directly proportional to the number of living cells. All samples and controls were compared with negative control to calculate the percentage of vital cells, using the following equation:

(1) $$\begin{array}{c} \text{Viab}\%=100\times \frac{\text{O}.{\text{D}}_{490\text{e}}}{\text{O}.{\text{D}}_{490\text{b}}}, \end{array}$$

where O.D_490e_ is the mean value of the measured optical density of extracts and O.D_490b_ is the mean value of the measured optical density of the negative control. A sample is considered cytotoxic if the percentage vitality value is <70% and noncytotoxic if the percentage vitality value is >70%.

### 2.5. Proliferation Test on the L929 Cell Line

Cells were seeded into ten 12.5 cm^2^ cell culture flasks at 4 × 10^4^ cells/ml in a total volume of 5 ml of extraction medium containing sample (0.2 g/ml). One flask containing only extraction medium was used as control (RC). Cells were maintained at +37°C ± 1 in a humidified 5% CO_2_ atmosphere and monitored daily by using an inverted microscope for 72 h. After 72 h of incubation, cells were trypsinized and counted by using a Burker camera and Trypan blue staining, in order to evaluate the proliferation rate.

### 2.6. Haematoxylin/Eosin Staining on the L929 Cell Line

Cells were seeded into 6-well culture plates at 1 × 10^5^ cells/ml ratio in extraction medium containing each sample. Cells were maintained at +37°C ± 1 in a humidified 5% CO_2_ atmosphere for 72 h in order to perform haematoxylin/eosin staining. Briefly, media were removed from each well and cells were washed with PBS and fixed in methanol; 1% haematoxylin (Sigma-Aldrich) solution was added, followed by PBS washings and 1% eosin staining (Sigma-Aldrich). Cell morphology was evaluated by using an inverted microscope supplied with a camera (Leica).

## 3. Results

**Figure 1:**
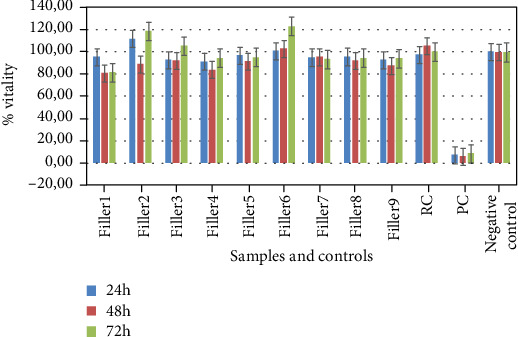
Effects of HA filler extracts and controls on L929 cells at 24 h, 48 h, and 72 h of exposure. Data express the percentage of cell viability.

Figure 1 shows results the calculation using the equation (1) for determining cell viability (MTS assay) of L929 exposed to the different extracts. The results of cell viability showed that none of the nine fillers analysed had cytotoxic effects on L929 cells at 24 h, 48 h, and 72 h, having viability values > 70%. However, filler 2 and filler 6 showed a higher cell viability percentage in comparison to other fillers tested and to the RC at 72 h, while filler 1 showed the lowest cell viability percentage at 72 h. Phenol solution 0.5% induced high levels of mortality (viability < 9%). Diluted samples induced no cytotoxicity; diluted phenol solution induced cytotoxicity until the 0.125% concentration (data not shown).

**Figure 2:**
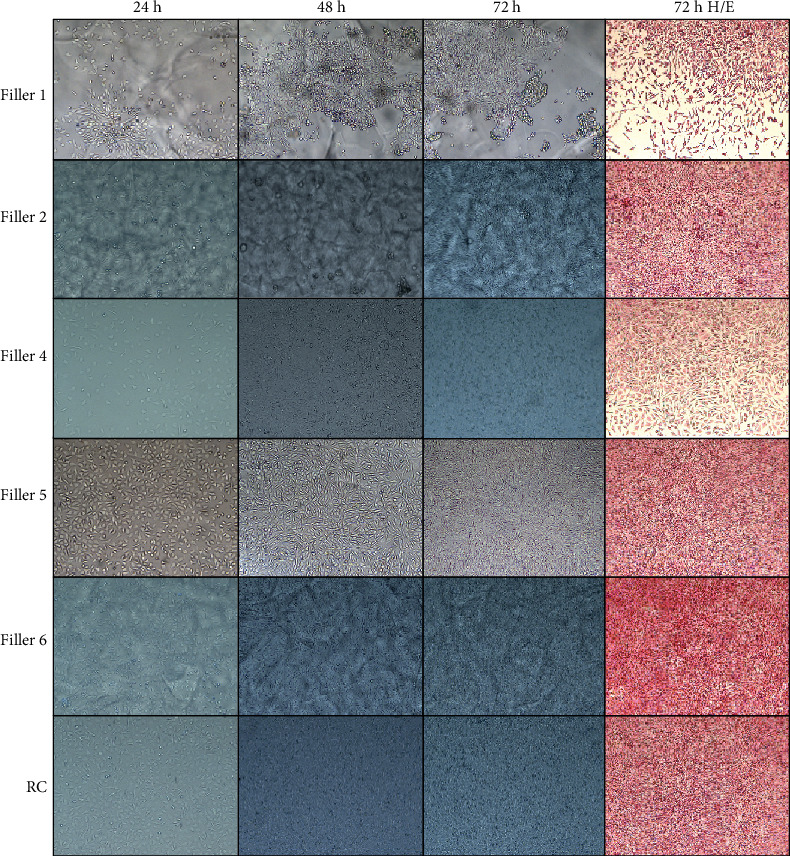
Morphologic evaluation of L929 cells exposed to different HA filler extracts. Observation conduced at 24 h (first column), 48 h (second column), 72 h (third column), and haematoxylin/eosin staining after 72 h (forth column). Magnification 100x.

Data obtained from the cell proliferation test are reported in Table 2. Filler 1 and filler 4 showed a lower cell number after 72 h grown; filler 2 and filler 6 showed an overgrowth of cells suggesting a proliferative effect of these samples on cells; the other samples showed a cell proliferation rate comparable to RC.  Figure 2 shows the morphology of L929 when cultured with filler 1 (low cell count), filler 2 (high cell count), filler 4 (low cell count), filler 5 (cell count similar to RC), filler 6 (high cell count), and RC for 24 h, 48 h, and 72 h. The last column shows cells stained with haematoxylin/eosin at 72 h. The other samples (fillers 3, 7, 8, and 9) showed a morphology and cell growth similar to RC (data not shown). Moreover, no micorbic growth was observed during the monitoring of cell cultures in contact with the fillers.

## 4. Discussion

In this work, authors report an *in vitro* study conducted on nine HA dermal fillers among the most commonly used in aesthetic medicine and dermatology, randomly received in the laboratory. According to the UNI EN ISO 10993 regulation, the L929 cell line was chosen for cytotoxicity assay, cell proliferation test, and cell morphology evaluation by haematoxylin/eosin staining. As demonstrated in a previous study, L929 cells are suitable to undergo a cytotoxicity test by MTS assay, as they are sensible to the reference materials indicated in the rule and able to well respond against any cytotoxic substance released in the culture media [12]. The MTS assay was chosen due to its low cost, accuracy, rapidity, and reproducibility. In addition, cells were cultured with fillers to evaluate the effects on cell growth in terms of cell count and morphology. These are two simple and rapid methods that, together with MTS assay, could improve the assessment of the biocompatibility of a material designed for human use [18].

Although all samples tested did not shown any cytotoxic effect on L929 cells (cell viability > 70%) after 24 h, 48 h, and 72 h, two of tested samples (fillers 2 and 6) showed a higher cell viability percentage compared to RC while other two samples (fillers 1 and 4) showed a lower percentage of viability compared to the same control (Figure 1). Moreover, the samples showed different effects on morphology of cell monolayers and on cell count. After 72 h of exposition of L929 to fillers, cell count was comparable to the untreated control (RC) for most of the samples, while fillers 1 and 4 showed a cell count reduced by about half and fillers 2 and 6 showed a significant increase in cell count. These results show that, although all examined fillers are not cytotoxic, some of them could have a different *in vitro* behaviour on cell proliferation by promoting or inhibiting it. These observations were confirmed during the daily monitoring of the monolayers by an optical microscope, since they reached the confluence in different times. Although data on the *in vivo* effect are not considered in this study, this different behaviour could lead to some *in vivo* implications in terms of effectiveness of treatment.

In recent years, the use of HA dermal fillers has widely spread in aesthetic medicine and dermatology [6, 19]. HA is a natural and biocompatible polymer that has a rapid turnover and is quickly degraded by enzymes. To produce a more resistant form of HA, it is cross-linked and stabilized using other substances. According to the cross-linking techniques, HA fillers are classified in monophasic or biphasic [20–22]. Results presented in this study demonstrate that the augment or decrease of the cell number does not depend on the nature of the filler (monophasic or biphasic). Injectable fillers are considered medical devices for which a control by the competent authorities is not required. However, it could be advisable to investigate about their *in vitro* effects in order to support their safety and to guarantee the absence of risks for the customer. According to the European Directive 63/2010/EU, standardized and validated *in vitro* methods could significantly contribute to limit the use of laboratory animals in biocompatibility tests. Therefore, in that contest, this study shows the suitability of some easy and rapid cell-based methods to assess the biocompatibility and the *in vitro* effects of HA fillers largely used in aesthetic medicine and dermatology and also predicts a possible *in vivo* effects in terms of safety and efficacy.

## 5. Conclusions

All HA fillers tested did not shown any cytotoxic effect on L929 cells (cell viability > 70%) after 24 h, 48 h, and 72 h. Two samples showed a higher cell viability percentage and two samples showed a lower percentage compared to RC. Differences were also found on morphology of cell monolayers and on cell count. These results show that, although all examined fillers are not cytotoxic, some of them could have different effects on cell proliferation and grown. The easy and rapid assays performed could be useful to test new materials, in terms of cytotoxicity and effects on cells, intended for human use without animal tests.

## Figures and Tables

**Table 1:** Characteristics of the nine HA fillers used.

Samples	Description
Filler 1	Stabilized hyaluronic acid 20 mg/ml, nonanimal origin, pH 6–7.5 biphasic
Filler 2	Cross-linked hyaluronic acid 20 mg/ml with lidocaine hydrochloride 3 mg/ml, biphasic
Filler 3	Hyaluronic biorevitalizing gel, medium-chain hyaluronic acid 20 mg/ml in a physiologic buffer, produced from *Streptococcus equi* bacteria
Filler 4	Hyaluronic acid 20 mg/ml-sodium chloride 16 mg/ml, from bacterial biofermentation, BDDE cross-linking agent, monophasic
Filler 5	Reticulated hyaluronic acid 25 mg/ml with lidocaine hydrochloride 3 mg/ml, animal origin, pH 7.2
Filler 6	Reticulated hyaluronic acid 24 mg/ml in physiological buffered saline, from biotechnological fermentation, low BDDE content, monophasic
Filler 7	Auto cross-linked hyaluronic acid–sodium chloride 18 mg/ml
Filler 8	Hyaluronic acid 2 mg and cross-linked HA 20 mg-sodium chloride 6.9 mg, nonanimal origin, endotoxin- and BDDE-free
Filler 9	Cross-linked hyaluronic acid 20 mg/ml-lidocaine hydrochloride 3 mg/ml pH 7, biphasic

**Table 2:** Cell count of L929 after 72 h exposure to each HA filler.

Samples	Cells/ml
Filler 1	530.00
Filler 2	1.355.000
Filler 3	1.150.000
Filler 4	650.000
Filler 5	1.005.000
Filler 6	1.555.000
Filler 7	900.000
Filler 8	1.145.500
Filler 9	850.000
RC	1.050.000

## Data Availability

The data used to support the finding of this study are available from the corresponding author.
